# Relationship between mid upper arm circumference and weight changes in children aged 6–59 months

**DOI:** 10.1186/s13690-015-0103-y

**Published:** 2015-12-21

**Authors:** Paul Binns, Nancy Dale, Monsurul Hoq, Chrissy Banda, Mark Myatt

**Affiliations:** Valid International, Oxford, UK; University of Tampere, Tampere, Finland; Terre des hommes, Dhaka, Bangladesh; Valid International, Lilongwe, Malawi; Brixton Health, Llawryglyn, UK

**Keywords:** Community-based management of acute malnutrition, CMAM, Mid upper arm circumference, MUAC, Severe acute malnutrition, SAM, MUAC gain, Weight gain

## Abstract

**Background:**

The objectives of this study were to (i) describe the relationship between weight changes and MUAC changes in children aged between 6 and 59 months during treatment for SAM in CMAM programmes in three country contexts (Malawi, Ethiopia and Bangladesh) admitted using MUAC and (ii) describe the sensitivity of both MUAC and weight to episodes of disease experienced during the SAM treatment episodes in CMAM programmes in three country contexts (Malawi, Ethiopia and Bangladesh) admitted using MUAC.

**Methods:**

Data collected under research conditions in Malawi were analysed for the correlation between MUAC and weight changes using the Pearson product–moment correlation coefficient (Pearson’s *r*). Further data from other CMAM programmes implemented under field conditions in Ethiopia and Bangladesh were similarly analysed. The association of growth failure following recent episodes of illness were assessed for MUAC and weight change using a two-by-two table, box-plots and Kruskal Wallis non-parametric rank sum test.

**Results:**

MUAC and weight gain acheived over the entire treatment episode were strongly correlated in all three country contexts, Ethiopia (median Pearson's r = 0.816, 95 % CI = 0.782 - 0.845), Malawi (median Pearson's r = 0.843, 95 % CI = 0.802 - 0.876) and Bangladesh (median Pearson's r = 0.725, 95 % CI = 0.663 - 0.777). MUAC and weight changes at each outpatient visit were closely correlated (median Pearson’s *r* = 0.954, 95 % CI = 0.602 – 0.997) under research conditions. The field data from Ethiopia and Bangladesh showed similar correlation (median Pearson’s *r* = 0.945, 95 % CI = 0.685 – 0.998) and (median Pearson’s *r* = 0.939, 95 % CI = 0.705 – 0.994) respectively. MUAC and weight appear to respond rapidly and similarly to episodes of illness reported during outpatient treatment for SAM for MUAC, diarrhoea RR = 1.88 (95 % CI = 1.64 - 2.15), vomiting RR = 1.89 (95 % CI = 1.58 - 2.26), fever RR = 1.57 (95 % CI = 1.36 - 1.82) and cough1.42 (95 % CI = 1.22 - 1.65). Similar relative risks are seen for weight; diarrhoea RR = 2.03 (95 % CI = 1.77 - 2.31), vomiting RR = 2.09 (95 % CI = 1.77 - 2.47), fever RR = 1.76 (95 % CI = 1.53 - 2.03) and cough RR = 1.25 (95 % CI = 1.06 - 1.48).

**Conclusions:**

This study demonstrates a close relationship between MUAC and weight change during recovery from SAM under both research and operational field conditions. Furthermore, changes in both MUAC and weight are observed to occur similarly and rapidly during episodes of illness occurring during treatment with no lag effect on the part of MUAC. This presents the possibility for children undergoing outpatient treatment for SAM to be monitored using MUAC as an alternative to weight. Further research would be required to develop a tool which can be deployed safely and enable MUAC to be used as the sole anthropometric measure for admission, monitoring of recovery and discharge. This development would potentially allow the further decentralisation of the treatment of SAM thus improving programme coverage and child survival.

## Background

In 2005, the World Health Organisation (WHO) endorsed the use of mid upper arm circumference (MUAC) as an independent admission criterion for use in selective-entry feeding programmes [1]. A MUAC greater than 125 mm has also been recommended as a discharge criterion [2] for such programmes and has been shown to be a safe criterion for discharge of children aged 6 to 59 months from outpatient treatment of severe acute malnutrition (SAM) [3, 4].

Current SAM treatment protocols recommend weight gain as the primary method of monitoring the recovery of children during treatment for SAM [5].

It has been suggested that MUAC is not suitable for monitoring recovery from SAM as increases in MUAC are thought to lag behind increases in weight:

“*Children classified as severely malnourished on the basis of low MUAC should be evaluated for recovery on the basis of anthropometric measures other than MUAC. MUAC may lag behind in recovery and may suggest non response to interventions if considered in isolation*” [6].

In December 2012, a consultation between United Nations, academic, and non-governmental organisation (NGO) staff responsible for the design and management of therapeutic feeding programs organised by the Emergency Nutrition Network (ENN) identified a lack of evidence regarding the relationship between weight gain and MUAC changes although recent studies had suggested a close relationship between the two measures [7].

This paper examines the relationship between weight gain and MUAC changes in children aged 6 to 59 months who were treated for SAM as outpatients in Community-based Management of Acute Malnutrition (CMAM) programmes in Malawi, Ethiopia and Bangladesh.

The objectives of this study were to:

1. Describe the relationship between weight gain and MUAC changes in children aged between 6 and 59 months during treatment for SAM in CMAM programmes in three country contexts (Malawi, Ethiopia and Bangladesh) admitted using MUAC.
2. Describe the sensitivity of both MUAC and weight to episodes of disease experienced during the SAM treatment episodes in CMAM programmes in three country contexts (Malawi, Ethiopia and Bangladesh) admitted using MUAC.

## Methods

In Malawi, data were collected on children aged 6 to 59 months with a MUAC ≤ 115 mm enrolled into outpatient treatment for SAM, following Malawi National CMAM Guidelines [8], at Ministry of Health facilities. MUAC and weight were measured on admission and at each subsequent weekly visit until they reached the discharge criteria of having a MUAC greater than 125 mm at two consecutive visits. Information on illness was recorded each week based on seven-day recall from the carer attending with the child. These data were collected under research conditions by a single observer to minimise measurement errors. Data were double-entered and verified using EpiData [9].

Prospective data were also obtained from CMAM programmes implemented by NGOs in two other country contexts: Ethiopia (Save the Children USA (SC-US)) and Bangladesh (Terre des homes (Tdh)).

Measurement and collection of the SC-US Ethiopia data were supervised by SC-US supervisors who were present at all clinic sessions. The SC-US Ethiopia data were entered from beneficiary record cards. Data were entered once using EpiData with interactive range and legal value checks applied.

Measurement and collection of the Tdh Bangladesh data were supervised by Tdh monitoring and evaluation staff who visited clinics on a rotating basis with each clinic being visited on, at least, a quarterly basis. Clinic activities were monitored using a checklist of observations of key activities. At each visit, a small sample (*n* = 5) of children had their records checked and anthropometric measurements retaken. If discrepancies were found in the records of measurements of more than one of five sampled children, then the clinic was marked for special measures (i.e. training and supervision visits and more intensive monitoring) until data-quality issues were resolved. The Bangladesh Tdh data were entered from beneficiary record cards. Data were entered once using SPSS Data Collection/Data Entry module (version 17) with interactive range and legal value and checks applied [10].

The data from all three countries were for children recruited sequentially into the respective programmes at several different programme sites. Only data for children with a MUAC of < 115 mm on admission was included in the present study. Although the programmes in Ethiopia and Bangladesh also enrolled children according to criteria using weight for height and oedema, exclusions for the purposes of data analysis included children with MUAC > 115 mm and children with oedema in order to make the data comparable from the three contexts. Other censored data included children with fewer than 3 visits, duplicate registration numbers and bizarre data (impossible measurements of weight or MUAC). All data were analysed irrespective of treatment outcome.

Analysis of the data and production of graphical figures was done using R Language for Data Analysis and Graphics [11].

Data were plotted and Pearson’s product–moment correlation coefficients (r) [12] calculated for the association between weight gain and MUAC gain achieved over the entire treatment episode:

1 $$ Weight\kern0.5em  Change\kern0.5em =\kern0.5em  Dicharge\kern0.5em  Weight\kern0.5em -\kern0.5em  Admission\kern0.5em  Weight $$

2 $$ MUAC\kern0.5em  Change\kern0.5em =\kern0.5em  Discharge\kern0.5em  MUAC\kern0.5em -\kern0.5em  Admission\kern0.5em  MUAC $$

Weight and MUAC at each visit were also plotted (i.e. as a time-series) and Pearson's product–moment correlation coefficients (*r*) calculated for the association between weight and MUAC at each visit for each treatment episode separately. Pearson’s r values are presented graphically and numerically as a median and 95 % CI.

Exemplars of graphical data were plotted, for the purpose of illustration to show the changes in MUAC and weight over the course of the treatment episode and with additional exemplar plots with episodes of reported illnesses indicated. The exemplars were selected purposively so as to be typical graphical representations of changes in MUAC and weight during recovery.

Histograms of Pearson’s r values were plotted for each dataset for all treatment episodes irrespective of the outcome of treatment.

The effect of a reported episode of illness in the seven days prior to a clinical visit on MUAC and weight change over the previous even days was assessed in two ways:

1. The relative risk of losing or failing to gain MUAC or weight over the previous seven days was estimated for all clinic visits subsequent to admission using a simple two-by-two table analysis (*n* = 1696 child-weeks).
2. The magnitude of MUAC or weight change over the previous seven days for children with and without a specific reported illness was examined using box plots and using a Kruskal-Wallis rank-sum test (a non-parametric method for testing whether samples originate from the same distribution).

## Results

Table 1 shows the characteristics of the study populations at admission and Table 2 describes the period of programme implementation, number of study subjects, admission criteria, data collection and discharge criteria of the study cohorts.

**Table 1:** Characteristics of study populations at admission

Ethiopia		n	%				
	Males	199	46.2 %				
	Females	232	53.8 %				
		Min.	Q1	Median	Mean	Q2	Max.
	Age at admission (months)	7.0	25.1	37.0	39.5	48.0	66.0
	MUAC at admission (cm)	7.5	10.2	10.5	10.4	10.8	10.9
	Height at admission (cm)	61.5	73.5	80.4	81.0	88.0	109.2
Malawi		n	%				
	Males	105	44.7 %				
	Females	130	55.3 %				
		Min.	Q1	Median	Mean	Q2	Max.
	Age at admission (months)	6.0	10.0	14.0	16.4	21.0	51.0
	MUAC at admission (cm)	8.2	10.5	11.0	10.8	11.4	11.5
	Height at admission (cm)	53.3	63.0	67.2	67.5	72.2	92.5
Bangladesh		n	%				
	Males	88	33.3 %				
	Females	176	66.7 %				
		Min.	Q1	Median	Mean	Q2	Max.
	Age at admission (months)	6.0	7.0	10.0	12.9	17.0	56.0
	MUAC at admission (cm)	8.5	11.1	11.3	11.2	11.4	11.4
	Height at admission (cm)	51.6	62.3	65.6	67.4	71.8	99.0

**Table 2:** Characteristics of the three country cohorts

Location	Agency	Program start date	Program end date	Program admission criteria	Program discharge criteria	Data collected at each visit^a^	Number of episodes
Ethiopia^b^	SC-US	16/09/03	06/05/04	MUAC < 110 mm	WHM ≥ 80 %^c^ and MUAC ≥ 110 mm	MUAC Weight	431
Malawi^d^	MoH	01/03/11	28/02/12	MUAC < 115 mm	MUAC ≥ 125 mm	MUAC Weight Morbidity	235
Bangladesh^b^	Tdh	11/06/13	20/08/14	MUAC <115 mm	MUAC ≥ 115 mm andWHZ ≥ −3^e^ and weight gain > 15 %	MUAC Weight	264

^a^Heights at admission and discharge were also recorded: ^b^Data collected in a service context: ^c^The NCHS reference was used; ^d^Data were collected as part of a research project: ^e^WGS reference was used

**Fig. 1:**
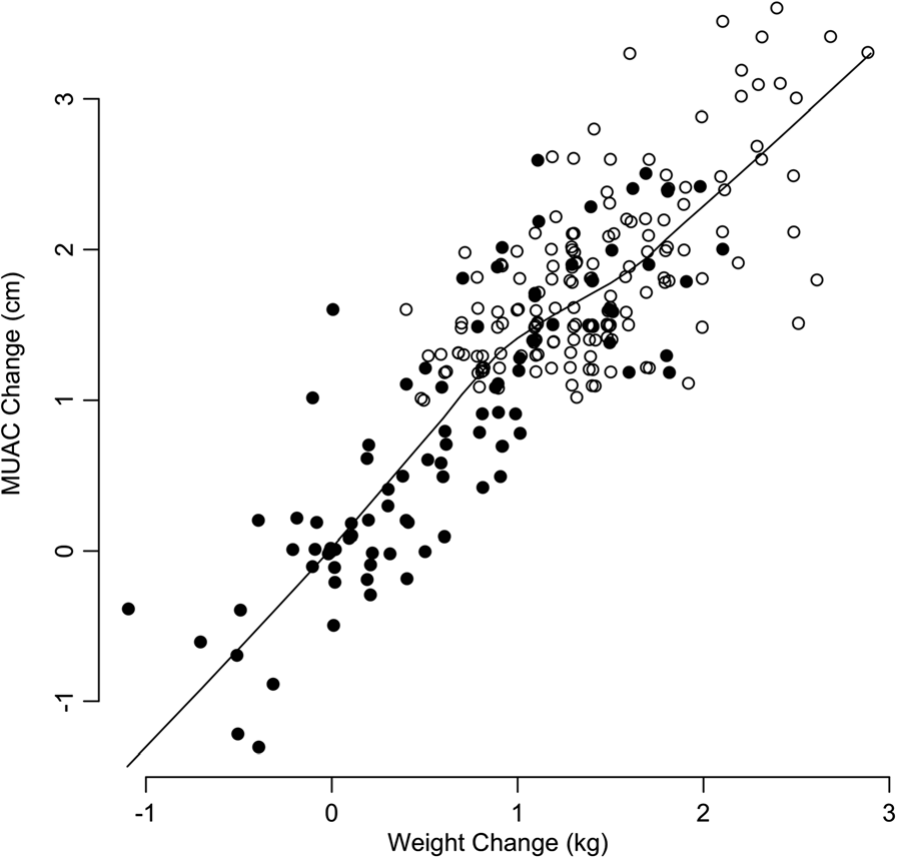
Change in MUAC (in cm) against change in weight (in Kg) for all treatment outcomes in the Malawi data set

Figure 1 shows the relationship between MUAC and weight changes observed in the Malawi data. Filled points in these plots are used to indicate negative outcomes (i.e. death, default, non-cured). Similar correlations in absolute MUAC and weight gains were observed in the data from Ethiopia and Bangladesh (see Table 3). MUAC gain and weight gain were strongly and positively correlated with each other. Recovery was associated with both higher weight gains and higher MUAC gains.

**Table 3:** Pearson’s product–moment correlation coefficients (r) calculated for the association between weight gain and MUAC gain achieved over the entire treatment episode

Country	Pearson's *r*
Ethiopia	0.816 (95 % CI = 0.782 - 0.845)
Malawi	0.843 (95 % CI = 0.802 - 0.876)
Bangladesh	0.725 (95 % CI = 0.663 - 0.777)

**Fig. 2:**
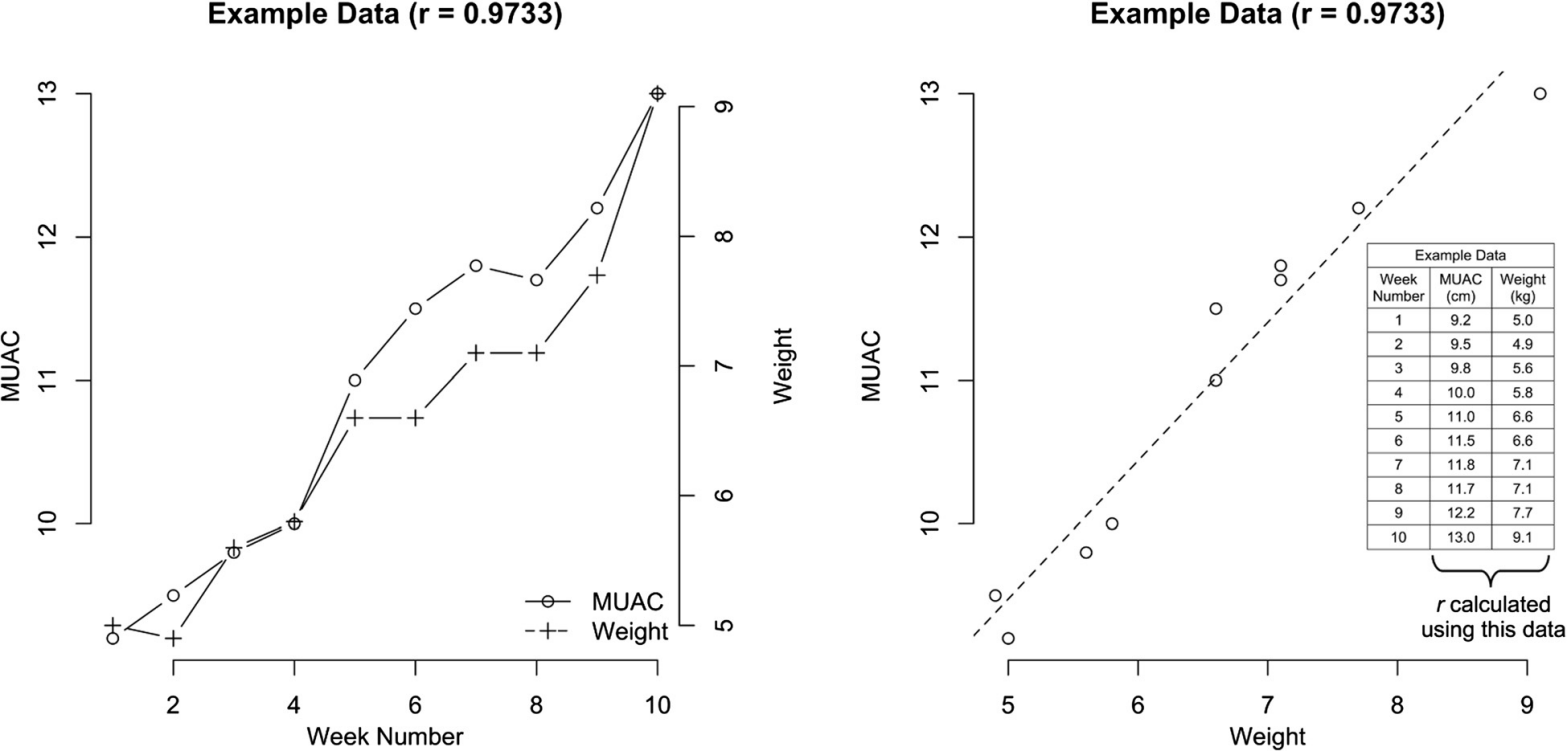
Example data and plots demonstrating the calculation of Pearson’s r correlation of MUAC and weight across the treatment episode

Figure 2 shows example data with the Pearson's correlation coefficients (r) calculated for the association between weight and MUAC at each visit for each treatment episode separately and as MUAC against weight for the same data in order to illustrate the methodology for the calculation of the correlation between MUAC and weight changes.

**Fig. 3:**
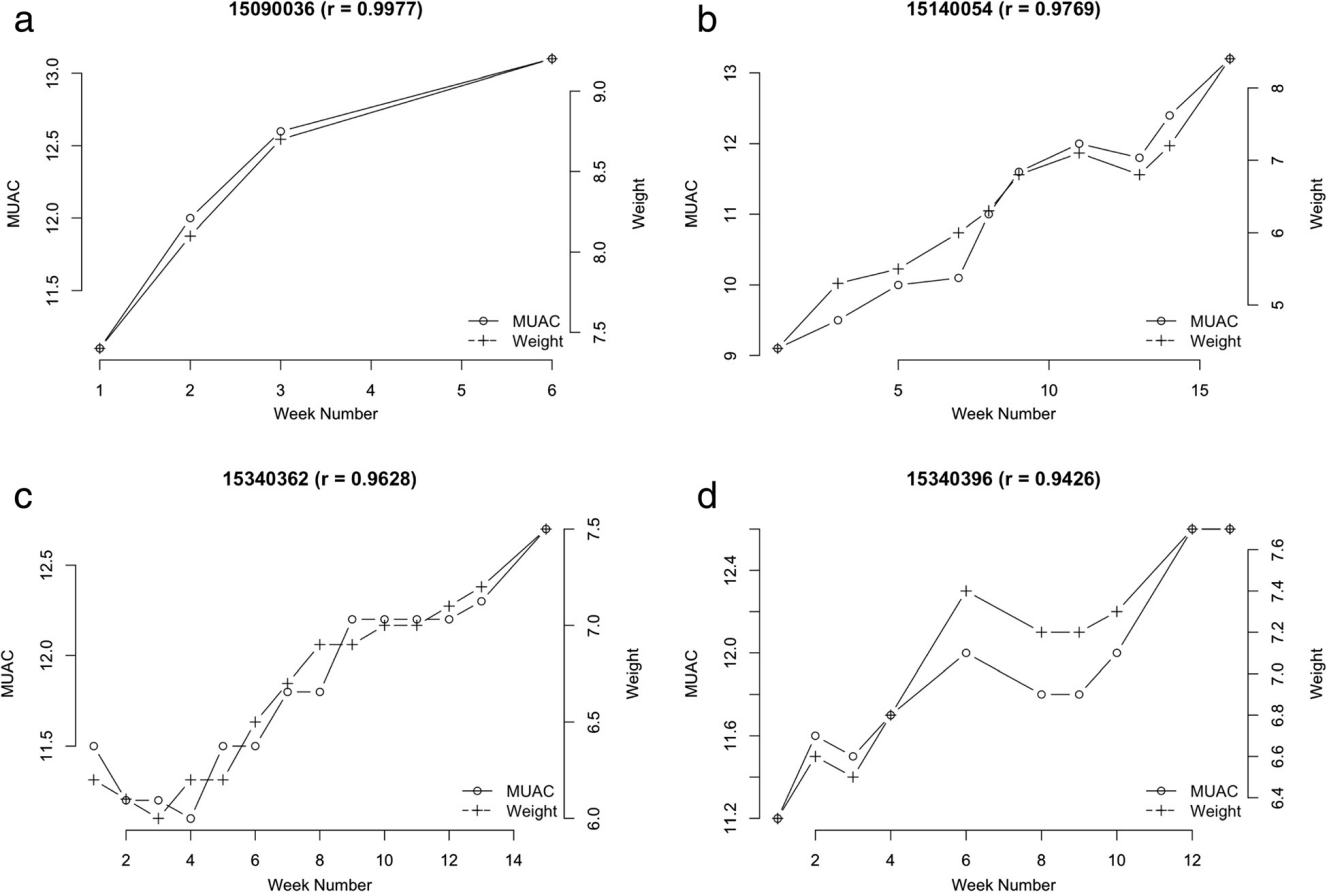
**a-d**: Exemplar graphic plots of MUAC and weight changes during recovery from SAM from Malawi data

**Fig. 4:**
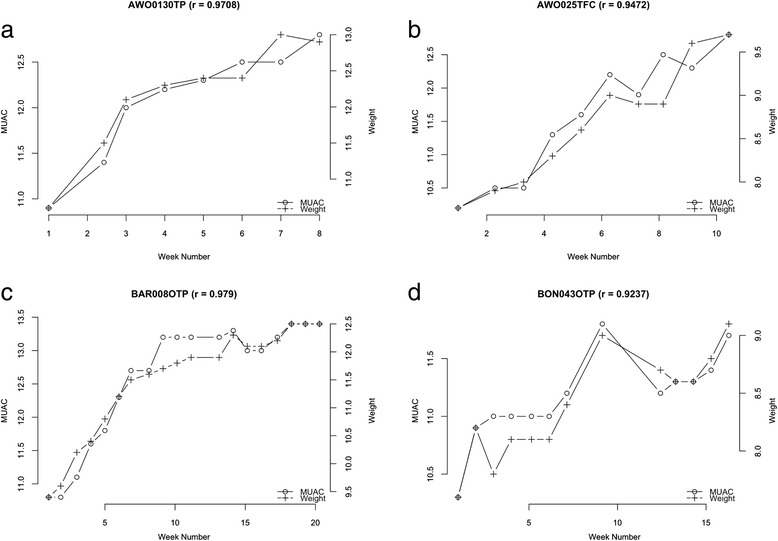
**a-d**: Exemplar graphic plots of MUAC and weight changes during recovery from SAM from Ethiopia data

**Fig. 5:**
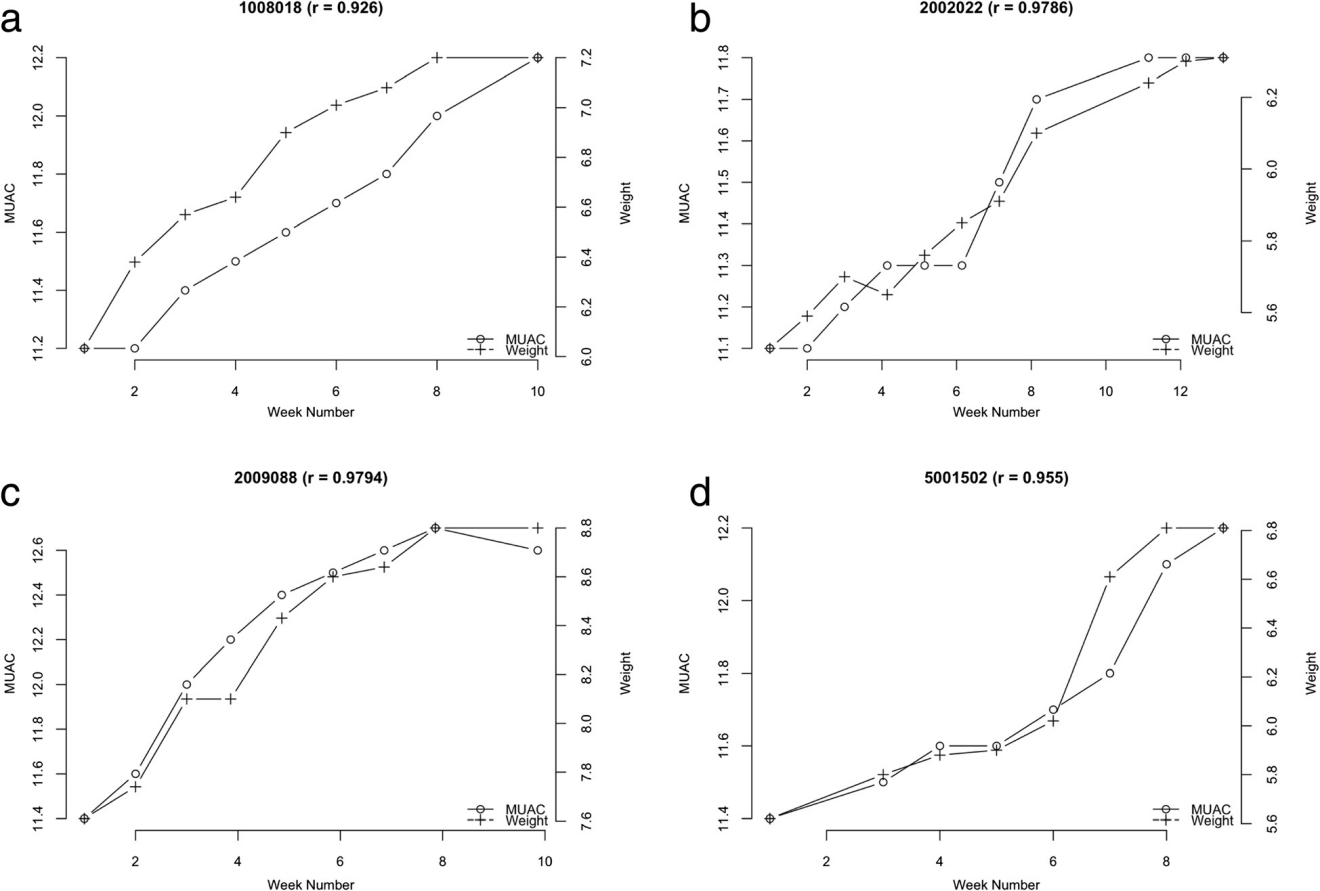
**a-d**: Exemplar graphic plots of MUAC and weight changes during recovery from SAM from Bangladesh data

The relationship between weight and MUAC changes at each visit for four typical exemplar cases undergoing treatment for SAM from each of the three country contexts is shown in the following figures for illustration purposes; Fig. 3a to d (Malawi), Fig. 4a to d (Ethiopia), and Fig. 5a to d (Bangladesh).

**Fig. 6:**
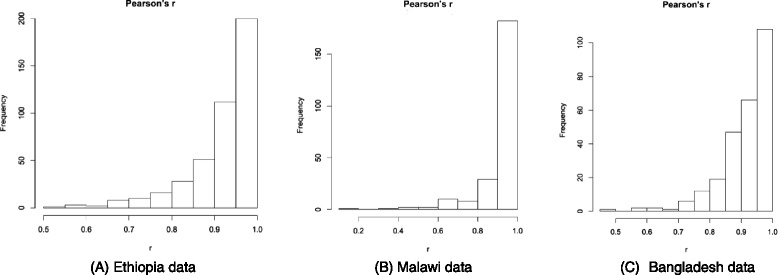
**a-c**: Histograms of Pearson’s r correlation for MUAC and weight for all treatment episodes irrespective of treatment outcome from Ethiopia, Malawi and Bangladesh

Figures 6a-c show histograms of the distribution of Pearson's correlation coefficient (r) for all data analysed for Ethiopia, Malawi and Bangladesh respectively, for all treatment outcomes. The median and 95 % CI of Pearson's correlation coefficient (r) for MUAC and weight changes occurring over the entire treatment episode for all cases is shown in Table 4. For all data sets there was a high correlation.

**Table 4:** Pearson's product–moment correlation coefficients (r) for weight and MUAC at each outpatient visit for all treatment episodes in the three country contexts

	Pearson's *r*	
Country	Median	95 % CI
Ethiopia	0.945	0.685 – 0.998
Malawi	0.954	0.602 – 0.997
Bangladesh	0.939	0.705 – 0.994

**Fig. 7:**
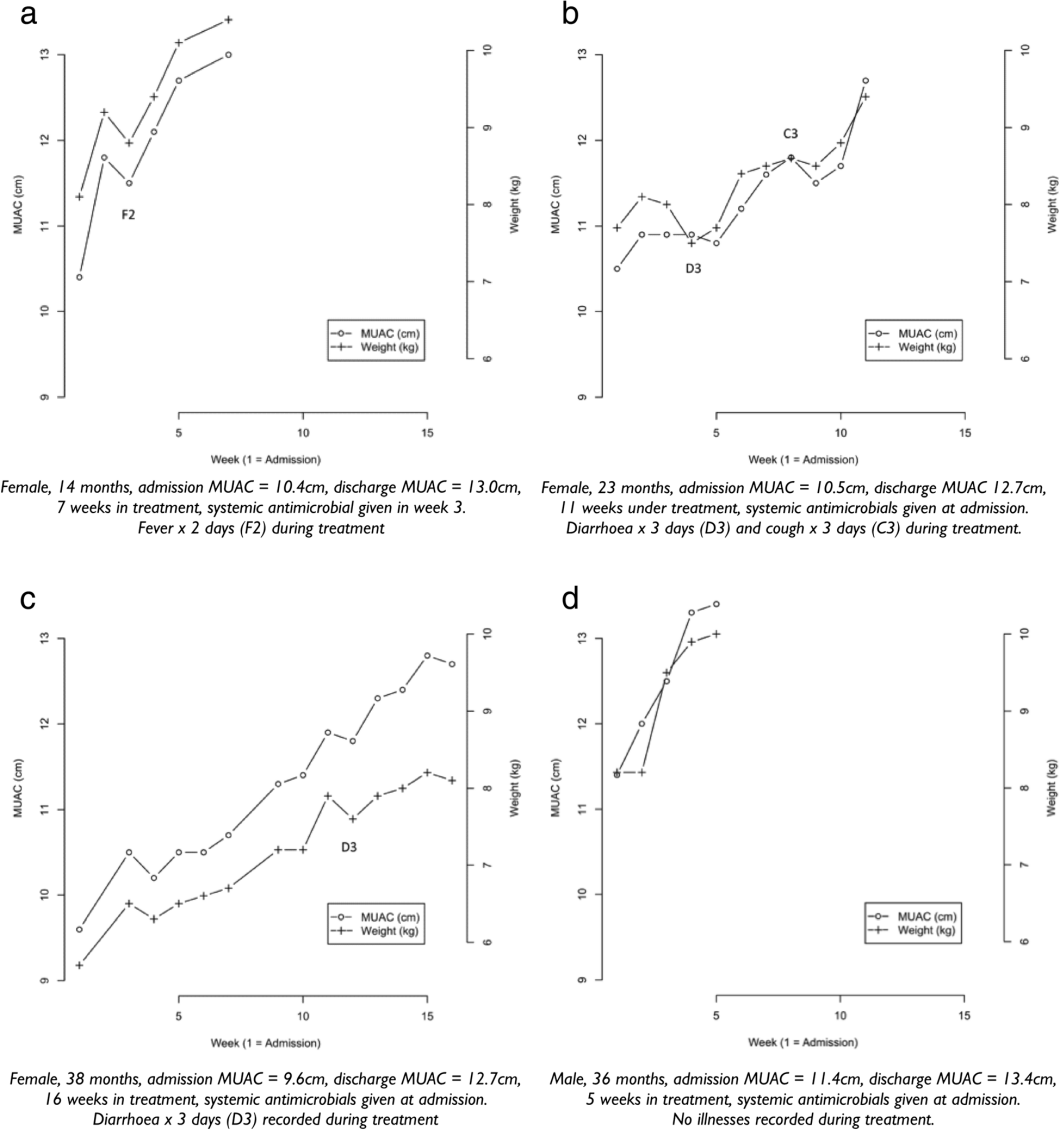
**a-d**: Exemplar graphic plots of MUAC and weight and their response to illness during recovery (taken from Malawi data)

Exemplar Fig. 7a to d show weight and MUAC at each visit with disease data plotted on each figure for the Malawi data. These illustrative figures show timing and duration in days of episodes of disease by maternal seven-day recall. Diseases are coded as C = cough, D = diarrhoea, V = vomiting, and F = fever. The number following the letter code is the duration of symptoms in days.

The relative risk (RR) of growth failure following any episode of reported illness against no reported illness are presented in Table 5. For illnesses involving fluid loss (diarrhoea, vomiting or fever), there is a similar 1.5 to 2 times risk of growth failure as measured by both MUAC and weight. The relative risk of growth failure following any episode of cough during the previous 7 days is similarly lower for both MUAC and weight (1.42, 1.25 respectively).

**Table 5:** Crude relative risk of growth failure subsequent to illness in the previous seven days

Reported exposure	MUAC	Weight
Any diarrhoea	1.88 (95 % CI = 1.64 - 2.15)	2.03 (95 % CI = 1.77 - 2.31)
Any vomiting	1.89 (95 % CI = 1.58 - 2.26)	2.09 (95 % CI = 1.77; 2.47)
Any fever	1.57 (95 % CI = 1.36 - 1.82)	1.76 (95 % CI = 1.53 - 2.03)
Any cough	1.42 (95 % CI = 1.22 - 1.65)	1.25 (95 % CI = 1.06 - 1.48)

**Fig. 8:**
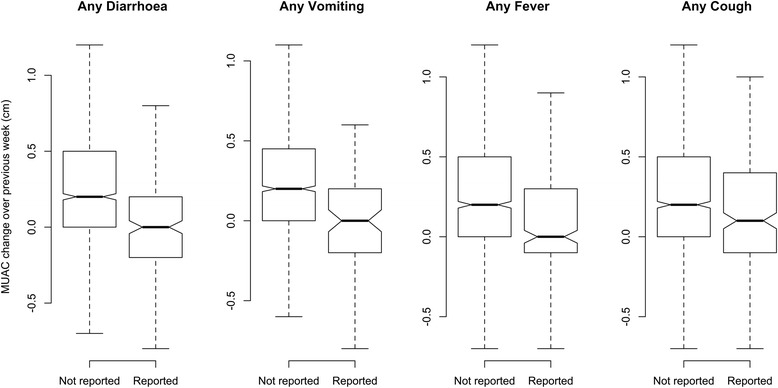
Box plot for change in MUAC in the previous seven days in response to any episode of reported illnesses

**Fig. 9:**
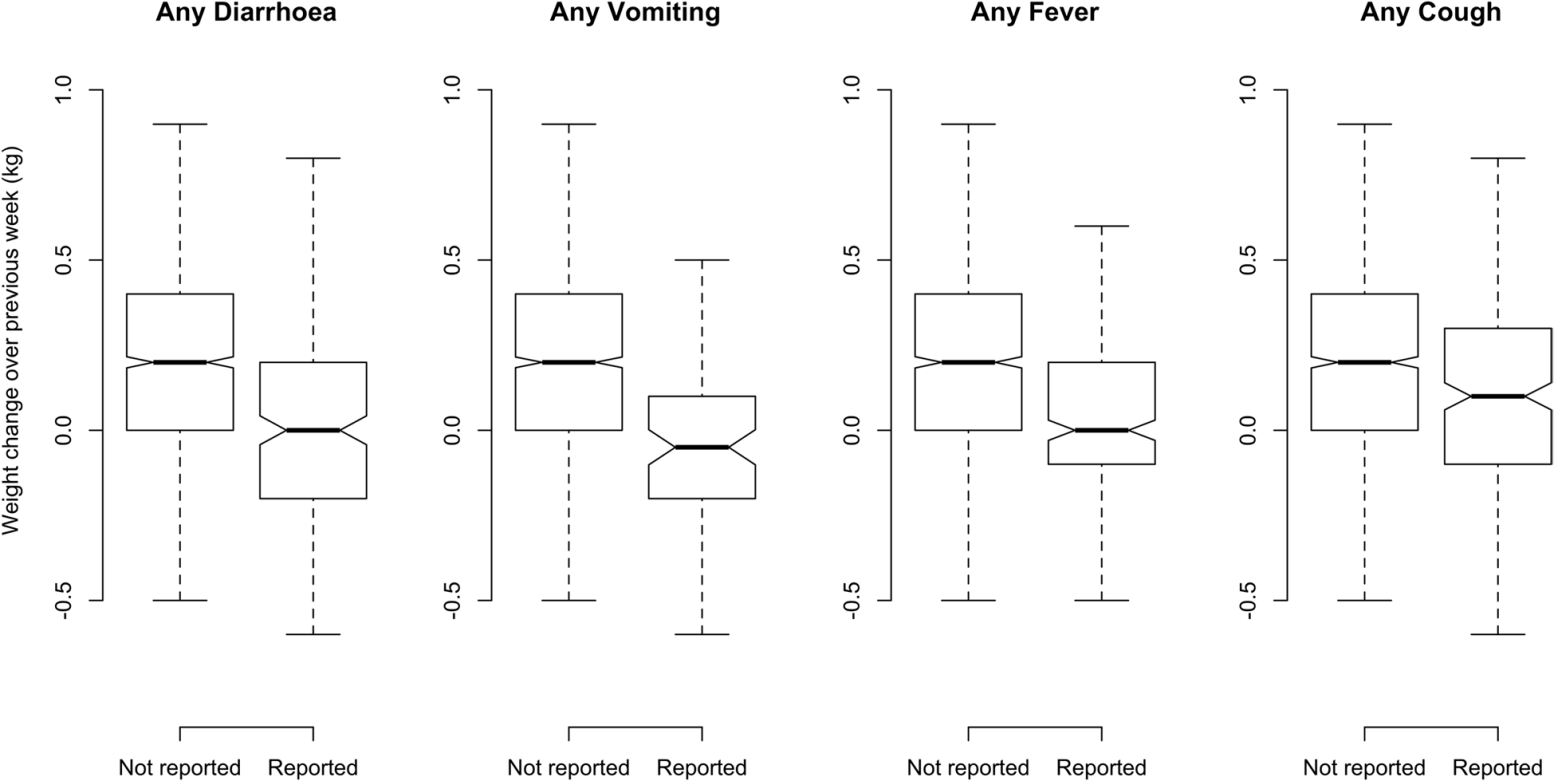
Box plot for change in weight in the previous seven days in response to any episode of reported illnesses

Figure 8 shows boxplots of changes in MUAC and Fig. 9 shows changes in weight, according to any episodes of diarrhoea, vomiting, fever or cough during the previous week reported by the caregiver against no reported illness. The boxplots show that MUAC and weight respond similarly and rapidly (i.e. within 7 days) to reported episodes of illness. The Kruskal-Wallis Chi-Square values for these changes are presented in Table 6 and are highly significant for all types of reported illness.

**Table 6:** Kruskal-Wallis rank-sum test for MUAC and weight change subsequent to illness in the previous seven days

	MUAC			Weight		
Reported exposure	Chi-Square	DF	p	Chi-Square	DF	p
Any diarrhoea	68.75	1	<0.0001	81.39	1	<0.0001
Any vomiting	33.82	1	<0.0001	41.64	1	<0.0001
Any fever	34.74	1	<0.0001	81.39	1	<0.0001
Any cough	15.00	1	0.0001	11.93	1	0.0006

## Discussion

The relationship between weight changes and MUAC changes in children 6–59 months at each follow up visit during outpatient treatment for SAM, shows a strong correlation in all contexts irrespective of the treatment outcome (see Tables 3 and 4 and Fig 6). This correlation was observed whether the data were collected under research or operational field conditions.

Both MUAC and weight changes respond negatively to episodes of illness with similar magnitudes of response for each illness. The response for both is similarly rapid (i.e. within a period of one week or less) with no obvious lag irrespective of the type of illness reported.

These findings are consistent with previously reported findings from rural Kenya [13].

The close correlation between changes in weight and MUAC with similar and rapid response to episodes of illness does not support the hypothesis that MUAC lags behind weight and is therefore an unsuitable measure for monitoring recovery.

With the acceptance of MUAC as both an admission and discharge criterion, the results of this study suggest that MUAC could also be used for monitoring the recovery of the child. While the response to treatment for SAM would normally be monitored using weight, the results of this study imply that a very similar recovery trajectory would be observed if MUAC were used for monitoring instead.

Programmes treating SAM cases as outpatients typically apply protocols which use weight for monitoring recovery where a lack of weight gain or weight loss may initiate actions such as follow up in the community or referral to hospital without a specific medical diagnosis being made. This study suggests that similar observations using MUAC may be also diagnostic of the need for intervention in a similar way. The lack of any lag effect and the similarity between weight and MUAC changes in response to illness would suggest that the diagnosis and responses to growth failure could be managed in an equally timely manner using MUAC.

From these data it can be postulated that if a child with SAM can be admitted, monitored during recovery and discharged safely using MUAC only, then this, with appropriate protocols to respond to observed negative changes in MUAC due to episodes of illness, could allow MUAC only treatment management. MUAC only case management could prove useful in expanding treatment coverage in locations where weighing equipment is either not available or cannot be maintained, or where barriers exist which prevent access to facility based treatment.

It is likely that tools used for monitoring MUAC and the associated protocols for appropriate referral and treatment will require literacy and numeracy skills similar to those used for completing growth charts. However, with field testing and refinement, it may be possible to develop simpler monitoring tools.

This potential for the increase in treatment coverage, through decentralisation of care from the health centre to community level, could improve the effectiveness of CMAM programming and thus improve child survival.

Limitations to this study include the exclusion of subjects admitted by weight for height and oedema criteria. Similar studies should be conducted in other contexts.

## Conclusions

Changes in weight and MUAC observed during treatment for SAM in outpatient therapeutic programmes are closely correlated in data from three different country contexts under research and field operational conditions. Changes in weight and MUAC resulting from episodes of diarrhoea, vomiting, fever or cough respond similarly and rapidly without any lag effect on the part of MUAC. This study suggests that monitoring of MUAC during treatment for SAM could provide a useful alternative to monitoring weight. Admission, monitoring recovery and discharge from treatment using MUAC alone provides a potential opportunity to further decentralise the treatment of SAM to areas where weighing equipment may be unavailable or access to health facilities is limited, potentially improving programme coverage and effectiveness. Further research is required in order to develop and test appropriate MUAC monitoring tools and safe corresponding care protocols for field testing in various contexts.

## Abbreviations

CMAM: Community-based management of acute malnutrition
ENN: Emergency nutrition network
MUAC: Mid upper arm circumference
NGO: Non Governmental Organisation
SAM: Severe acute malnutrition
SC-US: Save the children
Tdh: Terre des hommes
WHO: World Health Organisation
